# Cancer Incidence and Mortality in Swedish Sterilant Workers Exposed to Ethylene Oxide: Updated Cohort Study Findings 1972–2006

**DOI:** 10.3390/ijerph8062009

**Published:** 2011-06-03

**Authors:** Zoli Mikoczy, Håkan Tinnerberg, Jonas Björk, Maria Albin

**Affiliations:** 1 Division of Occupational and Environmental Medicine, Department of Laboratory Medicine, Lund University, SE-221 85 Lund, Sweden; E-Mails: hakan.tinnerberg@med.lu.se (H.T.); maria.albin@med.lu.se (M.A.); 2 Occupational and Environmental Medicine, Skåne University Hospital, SE-221 85 Lund, Sweden; 3 Competence Centre for Clinical Research, Lund University, SE-221 85 Lund, Sweden; E-Mail: jonas.bjork@skane.se

**Keywords:** cohort study, leukaemia, breast cancer

## Abstract

**Objectives:**

To assess whether cancer incidence, mainly from lymphohaematopoietic tumours and breast cancer, and mortality were increased in a cohort of Swedish sterilant workers exposed to low levels of ethylene oxide (EtO), updated with 16 more years of follow up.

**Methods:**

The mortality and cancer incidence 1972–2006 experienced by a cohort of 2,171 male and female workers employed for at least one year in two plants producing medical equipment sterilised with EtO were investigated. Individual cumulative exposure to EtO was assessed by occupational hygienists. Cause-specific standardized rate ratios were calculated using the regional general population as a comparison for mortality (SMR) and cancer incidence (SIR). Internal Poisson-regression analyses were performed for selected causes.

**Results:**

The median cumulative exposure to EtO was 0.13 ppm-years. The overall cancer incidence was close to unity (SIR 0.94, 95% CI 0.82–1.08). Eighteen cases of lymphohaematopoietic cancer were observed (SIR 1.25, 95% CI 0.74–1.98). A healthy worker effect was indicated from a significantly decreased overall mortality and mortality from cardiovascular diseases. Internal analyses found significantly increased rate ratios for breast cancer for the two upper quartiles of cumulative exposure as compared to the lowest 50% of the cohort (IRR 2.76, 95% CI 1.20–6.33 and IRR 3.55, 95% CI 1.58–7.93).

**Conclusions:**

The findings from this updated study indicate limited or low risks for human cancer due to occupational exposure from ethylene oxide at the low cumulative exposure levels in this cohort. However a positive exposure-response relation with breast cancer was observed though.

## 1. Introduction/Background

Ethylene oxide (EtO) has been produced since the beginning of the 20th century, and the annual world production is around seven million tons [1]. In Sweden EtO is produced on a large scale and mainly used as an intermediate in the chemical industry. EtO is also used as a sterilising agent for medical equipment. This was the main source of occupational exposure to EtO in Sweden, though nowadays the levels of exposure must be considered to be low and the exposed workers probably to be few. In an international perspective EtO is still used in large scale and the exposure has been described in some recent studies [2–6].

The International Agency for Research on Cancer (IARC) has classified EtO as carcinogenic to humans [7]. The evaluation was based on a combination of mechanistic data and sufficient evidence for carcinogenicity in experimental animals, while the evidence for carcinogenicity in humans was assessed as limited. This evaluation was reaffirmed by IARC in 2009 [8].

Inhalation of ethylene oxide causes cancer at multiple sites in mice (lung tumours, malignant lymphomas, uterine adenocarcinomas, and mammary carcinomas [9]), and in rats (leukaemia, brain tumours, mesotheliomas of the testis, and subcutaneous fibromas [10]). A large epidemiologic study of 18,000 employees at U.S. sterilant facilities found no overall increase in mortality from lymphohaematopoietic tumours [11]. Associations were, however, observed between cumulative exposure and mortality from non-Hodgkin’s lymphoma, multiple myeloma, and chronic lymphatic leukaemia in men. Other cohort studies have reported weak and non-significant risks for these tumours [12,13]. A significantly increased breast cancer incidence among EtO exposed female sterilant workers has been reported [14], and an exposure—response relation between EtO exposure and breast cancer incidence was observed [15]. In contrast, some recent studies report low or no exposure related effects [16,17].

The aim of the present study was to assess whether cancer incidence, especially from lymphohaematopoietic tumours and breast cancer, and mortality were increased at the low exposure levels experienced by the previously reported Swedish sterilant workers cohort [13], updated with 16 more years of follow up.

## 2. Methods

### 2.1. Cohort and Plants

The cohort comprised 2,171 workers (1,309 females and 862 males), employed for at least one year prior to 1986, at two plants producing medical equipment sterilised with EtO. Vital status in the cohort was determined on 31 December 2006 (Table 1).

In plant A the sterilisation operations started in 1970 and ceased in 1994. The initial exposures in 1970 could be as high as 40 ppm. Thereafter, the levels have continuously decreased, and since 1985 only sterilisers have had eight hour time-weighted exposures exceeding 0.2 ppm. Plant B started sterilising in 1964, and the process ceased in 2002. With initial peak exposure levels of 75 ppm in 1964 plant B has also had a continuous decrease of exposure, and since 1985 only sterilisers and some store workers have had eight hour time-weighted exposures >0.2 ppm.

### 2.2. Exposure Assessment

A detailed exposure assessment including two plant specific job-exposure matrices was performed for exposures up to 1986 [18]. All workers that were still employed at the end of this earlier follow up were listed (n = 1,303), and the lists were distributed to the two companies. They added the work histories for these workers until 1994 (plant A) and 2002 (plant B) when the use of EtO ceased. Results from the yearly statutory hygienic measurements of EtO from 1986 and on, were retrieved. The exposures for the 1303 workers were assessed on the basis of the old job exposure matrices and the more recent measurements. The impact of the updated exposure on the cumulative EtO-exposure (Table 2) was low, as expected. An addition to the cumulative EtO-exposure was seen for 145 (11%) of the 1,303 workers, with changes to the cumulative EtO-exposure in the range of 0.01–3.7 ppm-years. The greatest impact was on the 90th percentile which was changed from 1.17 to 1.29 ppm-years.

### 2.3. Information on Causes of Death and Tumours and Risk Estimates

Information on cause of death for the period 1972–2006, coded according to the International Classification of Diseases (ICD), 8th revision, was provided by Statistics Sweden, and on malignant tumours for the period 1972–2006 (coded according to ICD, 7th revision) from the Swedish Cancer Registry. We obtained information on vital status and date of emigration as of December 31, 2006, from the Swedish population registry. One hundred and seventy one of the 2,171 workers were deceased, 126 had emigrated and the rest were still alive and living in Sweden. None was lost to follow up.

Expected mortality and cancer incidence for the same period for the county of Scania was calculated by means of the SYDCAP cohort program using cause-, calendar year, sex, and 5-yr age group-specific rates. These rates were calculated from incidence rates for specific causes-of-death and malignant tumours and population counts, obtained from Statistics Sweden and the Swedish Cancer Registry. End of observation period, date of death, or date of emigration was used as individual endpoint for mortality, whichever occurred first. For cancer incidence, date of second tumour diagnosis, was included as potential individual endpoint. Altogether the cohort contributed 58,305 person-years under risk. The 95% confidence intervals (95% CIs) for cause-specific standardised mortality and incidence ratios (SMRs and SIRs) were calculated by treating the observed number as a Poisson variable, or as a normal variable if the observed value was greater than 15. The term significant indicates that the 95% CI does not include 1.00.

Expected mortality and cancer rates were calculated for the whole observation period 1972–2006, as well as for the earlier follow up period 1972–1990 which has been reported before [13], and the updated follow up period 1991–2006. Minor discrepancies in the expected numbers for the earlier follow up period may occur due to update of the software.

The extended follow up permitted us to use a 15 year induction latency period, rather than the 10 years used in the previous update [13]. Internal analysis of cancer incidence (Incidence rate ratio, IRR) between exposure categories within the cohort were performed by means of Poisson regression [19] using EGRET software (Statistics and Epidemiology Research Corporation, Seattle). Analyses were adjusted for gender, age (0–49, 50–59, 60–69 & ≥70) and calendar period (1972/1976–1979, 1980–1989, 1990–1999 & 2000–2006) with no induction latency period. Internal analyses were focused on total cancer incidence and diagnoses with a priori observed associations with EtO exposure, *i.e.*, breast cancer and lymphohaematopoietic cancers.

## 3. Results

### 3.1. Cancer Incidence

The SIR for overall cancer incidence in the external comparisons was close to unity, with 203 observed malignant tumours, compared with 216 expected (SIR 0.94, 95% CI 0.82–1.08) (Table 3). Eighteen cases of lymphohaematopoietic cancer were observed (SIR 1.25, 95% CI 0.74–1.98). Out of those five cases were leukaemia (SIR 1.40, 95% CI 0.45–3.26), nine non-Hodgkin’s lymphoma (SIR 1.44, 95% CI 0.66–2.73), one Hodgkin’s lymphoma and two multiple myelomas. Non-significantly enhanced rates were observed for cancer in the oesophagus, rectum, pancreas, cervix, urinary bladder, or brain.

Based on 13 cases an unexpectedly high SIR was observed for rectal cancer (SIR 1.68; 95% CI 0.89–2.86), with 12 of the cases occurring during the extended follow up 1991–2006 (SIR 1.92; 95% CI 0.99–3.35).

Comparison of the risk patterns for the two follow up periods, 1972–1990 which has been reported before [13] and 1991–2006, showed a somewhat higher estimate for all sites for the latter (SIR 0.98, 95% CI 0.83–1.14) than the earlier one (SIR 0.81, 95% CI 0.58–1.10; Table 4). However, during the latter observation period 12 new cases of lymphohaematopoietic tumours were observed, compared with 10.8 expected, giving a lower estimate (SIR 1.11, 95% CI 0.57–1.94) than for the earlier follow up (SIR 1.68, 95% CI 0.61–3.65). The same pattern was observed for leukaemia and multiple myeloma. When applying a 15 year induction latency period, the overall observed cancer incidence was similar, but the incidence of lymphohaematopoietic cancer was somewhat lowered.

In an analysis including only those with a cumulative EtO exposure above the median value (0.13 ppm-years), and applying a 15 year induction latency period, the incidence ratio for all sites was slightly increased, but still non-significant (SIR 1.06, 95% CI 0.86–1.28). This was also the case for breast cancer (SIR 1.20, 95% CI 0.78–1.75), and cervical cancer (SIR 1.95, 95% CI 0.40–5.69; based on three cases), while the rates for lymphohaematopoietic cancer, were somewhat decreased. Further restriction to cumulative exposure above the 75th percentile (0.22 ppm-years), increased the point estimate for breast cancer (SIR 1.41, 95% CI 0.82–2.26).

Internal comparison by cumulative exposure (Table 5) revealed higher, but non-significant total cancer incidence in the two highest quartiles of cumulative exposure (IRR 1.37, 95% CI 0.95–1.97 and IRR 1.34, 95% CI 0.94–1.90 respectively). This was not observed for lymphohaematopoietic cancer. However, significantly increased rate ratios for breast cancer were observed for the two upper quartiles of cumulative exposure as compared to the lowest 50% of the cohort (IRR 2.76, 95% CI 1.20–6.33 and IRR 3.55, 95% CI 1.58–7.93). Internal analysis dichotomized by median of duration of employment (12–68 months *vs.* ≥69 months) showed similar results with IRR 1.37 (95% CI 1.01–1.86) for total cancer, and IRR 2.75 (95% CI 1.32–5.72) for breast cancer, while no such pattern was seen when dichotomizing by median of exposure intensity.

### 3.2. Mortality

The overall mortality was significantly decreased (SMR 0.84, 95% CI 0.72–0.98) (not shown in table). This was also the case for deaths from cardiovascular diseases (SMR 0.69, 95% CI 0.50–0.94), which comprise 25% of all deaths in the cohort. A slight but non-significant increase was found for deaths from all malignancies (SMR 1.12, 95% CI 0.89–1.40). Based on 10 cases of deaths from pancreatic cancer an enhanced rate was seen (SMR 2.05, 95% CI 0.98–3.78). Numerical but non-significant increases of deaths from leukaemia, lymphoma and lung cancer were observed in the whole cohort, while slight but non-significant decreases were found for deaths from breast cancer, respiratory diseases and violent deaths. Point estimates (SMRs) for deaths from cancer of the pancreas and of the lung were higher among female workers, as compared to male workers.

Applying a 15 year induction latency period enhanced the point estimate for the overall mortality, which no longer was significantly below unity (SMR 0.87, 95% CI 0.73–1.03). Thirty-five of the 43 deaths from cardiovascular diseases still remained, and the rate was still significantly lowered (SMR 0.69, 95% CI 0.48–0.96). The SMR for deaths from pancreatic cancer increased significantly (SMR 2.24, 95% CI 1.02–4.25). The rates for deaths from lung cancer and breast cancer were somewhat increased, while the estimates for deaths from leukaemia, lymphoma and respiratory diseases, were lowered, all of them remaining non-significant.

Restriction to workers with a cumulative exposure above the median value (0.13 ppm-years), and applying a 15 year induction latency period, still showed a low mortality from all causes (SMR 0.80, 95% CI 0.63–0.99). A low risk was also seen for deaths from cardiovascular diseases (SMR 0.60, 95% CI 0.37–0.91). Higher than expected rates were seen for deaths from breast cancer, pancreatic cancer and leukaemia, while slightly decreased point estimates for deaths from lung cancer, and respiratory diseases were observed, none of which were statistically significant.

## 4. Discussion

Adding 16 years of follow up to the cohort, the incident cancer cases increased almost six-fold, the number of deaths was five-fold higher, and the person years under risk more than doubled. However the cohort is still relatively young and the power for rare tumours such as lymphohaematopoietic ones is still not satisfactory.

Significantly decreased overall mortality and mortality from cardiovascular diseases indicated a healthy worker effect. Observed overall mortality has been similarly low in other comparable cohorts [11,16]. Internal comparison may be indicated when such a bias occurs, thus Poisson regression analyses were performed for selected outcomes.

The cohort as a whole had very low cumulative exposure to EtO, as compared to some recently studied cohorts [11,16]. Only about 10% of the cohort had a minimum of 1.0 ppm-years. On the other hand a vast majority of the cohort (95%) had individual data on cumulative exposure. The update of the EtO exposure in the cohort marginally changed the cumulative EtO exposure for a small part of the cohort (7%).

The extended follow up permitted us to use an induction latency period of 15 years, as opposed to the 10 years used in the previous update of the cohort. The risk estimates are however similar (results not reported) whichever of the induction latency periods are chosen.

Since the previous update of the cohort the incident cases of lymphohaematopoietic cancer has increased from 6 to 18 cases, and three new cases of leukaemia had been diagnosed. However, the point estimates for both groups decreased to close to unity. Thus, this new follow up (including internal comparisons) did not detect any excess risk from lymphatic and haematopoietic cancer at these low levels of exposure.

Associations between EtO exposure and breast cancer incidence have been reported [14,15]. In our study the incidence of breast cancer, as well as the mortality from breast cancer, was increased when restricting the analysis to female workers with a cumulative EtO exposure above the median value of the cohort (0.13 ppm-years). An even higher incidence ratio for breast cancer was observed in a similar analysis restricted to workers in the upper quartile of EtO exposure (above 0.21 ppm-years). Internal direct comparison by cumulative EtO exposure showed higher incidence rate ratios for the two highest quartiles of exposure. Even though the two upper quartiles still include mainly very low exposures, this supports the previous findings. However, the associations seemed to be driven more by duration of employment than intensity of exposure. Moreover, previous associations were observed for substantially higher exposures [15] (median 8.4 and 14 ppm-years respectively for non-cases and cases).

We did unfortunately not have information available on reproductive history, BMI or life style factors which are of importance for breast cancer risk [20]. Even though substantial confounding in occupational epidemiology is rare [21], and internal analyses are likely to reduce such systematic differences between exposure groups, it may still be present. Shift work occurred at both plants, but has overall been associated with only low to moderate excess of breast cancer risk [22].

The discrepancy between the observed 10 deaths from pancreatic cancer, and the six incident pancreatic diagnoses, might be due to the fact that in some cases no pathological examination is available and the tumour may thus not be registered as a primary pancreatic tumour (personal communication from the Regional Tumour Registry). There might thus be an underestimate of the incident cases of pancreatic cancer. The elevated SIR found for rectal cancer was unexpected and has not been observed or reported from other occupational cohort studies of EtO.

The findings from this study speaks for limited or low risks for human cancer due to occupational exposure to ethylene oxide in the low cumulative exposure window covered by this study. However, a positive exposure-response relation with breast cancer was observed in internal analyses, which give some support to similar findings from earlier studies.

## Figures and Tables

**Table 1.** Vital status, as of 31 December 2006, in the cohort of 2171 workers exposed to EtO.

**Vital status**	**Plant A n (%)**	**Plant B n (%)**	**Total n (%)**
Living	1,003 (87.1)	871 (85.5)	1,874 (86.3)
Dead	75 (6.5)	96 (9.4)	171 (7.9)
Emigrated	74 (6.4)	52 (5.1)	126 (5.8)

Total	1,152 (100)	1,019 (100)	2,171 (100)

**Table 2.** Descriptive data for the Swedish sterilant workers cohort, as of 31 December 2006, divided into categories of cumulative EtO-exposure.

	***Cumulative exposure 0–0.13 ppm-years***	***Cumulative exposure 0.14–0.21 ppm-years***	***Cumulative exposure ≥0.22 ppm-years***	Cohort members with cumulative exposure *	***Cohort members employed ≥1 year***

	**Mean (Median)**	**Mean (Median)**	**Mean (Median)**	**Mean (Median)**	**Mean (Median)**
Age (at end of follow up)	52.4 (51)	58.8 (58)	60.6 (60)	55.9 (55)	55.7 (55)
Start of employment	1979 (1980)	1977 (1976)	1973 (1974)	1977 (1978)	1977 (1978)
Total employment (years)	3.58 (3.33)	8.33 (8.51)	11.5 (10.9)	6.5 (6)	6.3 (5.7)
Cumulative exposure to EtO	0.072 (0.07)	0.17 (0.17)	11.6 (0.39)	2.92 (0.13)	
Average exposure intensity (ppm)	0.0203 (0.02)	0.0206 (0.0202)	1.11 (0.1005)	0.2877 (0.0202)	
Gender Males (n)	424	199	200	823	862
Females (n)	615	287	295	1,197	1,309
Total (n)	1,039	486	495	2,020	2,171
Plant A (n)	524	326	266	1,116	1,152
B (n)	515	160	229	904	1,019
Total (n)	1,039	486	495	2,020	2,171

* Cumulative exposure could not be retrieved for 151 subjects due to lacking information on job titles.

**Table 3.** Cancer incidence 1972 *–2006 in EtO exposed workers employed for at least one year.

	
		**No induction latency period (n = 2,171) (person-years = 58,220)**				**≥15 years induction latency period (n = 2,046) (person-years = 27,415)**			

***Tumour site***	***ICD-7***	***O***	***E***	***SIR***	***95% CI***	***O***	***E***	***SIR***	***95% CI***
Oesophagus	150	3	1.63	1.84	0.38–5.38	3	1.40	2.14	0.44–6.26
Stomach	151	3	3.72	0.81	0.17–2.36	3	2.73	1.10	0.23–3.21
Colon	153	9	11.7	0.77	0.35–1.47	8	9.27	0.86	0.37–1.70
Rectum	154	13	7.76	1.68	0.89–2.86	12	6.20	1.94	1.00–3.38
Pancreas	157	6	3.79	1.58	0.58–3.45	4	2.94	1.36	0.37–3.48
Lung	162	17	15.1	1.13	0.66–1.80	15	12.3	1.23	0.69–2.02
Breast	170	41	50.9	0.81	0.58–1.09	33	38.54	0.86	0.59–1.20
Cervix	171	8	6.38	1.25	0.54–2.47	4	3.10	1.29	0.35–3.30
Prostate	177	17	15.3	1.11	0.65–1.78	16	14.24	1.12	0.64–1.82
Urinary organs, including bladder	181	11	8.02	1.37	0.68–2.45	9	6.60	1.36	0.62–2.59
Melanoma	190	8	12.7	0.63	0.27–1.25	5	8.11	0.62	0.20–1.44
Nervous system, including brain	193	11	8.46	1.30	0.65–2.33	7	5.35	1.31	0.53–2.70
Non Hodgkin’s lymphoma	200,202	9	6.25	1.44	0.66–2.73	7	4.68	1.50	0.50–3.08
Hodgkin’s lymphoma	201	1	1.31	0.76	0.02–4.25	0	0.54	0.00	0.00–6.83
Multiple myeloma	203	2	2.08	0.96	0.12–3.47	1	1.71	0.58	0.01–3.26
Leukaemia	204–205	5	3.58	1.40	0.45–3,26	3	2.60	1.15	0.24–3.37
Lymphohaematopoietic cancer	200–209	18	14.4	1.25	0.74–1.98	11	10.39	1.06	0.53–1.89
All sites	140–209	203	216	0.94	0.82–1.08	159	162	0.98	0.84–1.15

* The follow up period started 1976 for the workers from plant A.

**Table 4.** Cancer incidence 1972 *–2006 in EtO exposed workers employed for at least one year, divided by follow up period. No induction latency period was applied.

		Follow up period = 1972–1990 * (n = 2,171) (person-years = 26,674)				**Follow up period = 1991–2006 (n = 2,077) (person-years = 31,546)**			

***Tumour site***	***ICD-7***	***O***	***E***	***SIR***	***95% CI***	***O***	***E***	***SIR***	***95% CI***
Oesophagus	150	0	0.12	0.00	0.00–30.7	3	1.41	2.13	0.44–6.22
Stomach	151	0	0.95	0.00	0.00–3.88	3	2.78	1.08	0.22–3.15
Colon	153	3	2.31	1.30	0.27–3.80	6	9.34	0.64	0.24–1.40
Rectum	154	1	1.51	0.66	0.02–3.69	12	6.25	1.92	0.99–3.35
Pancreas	157	2	0.86	2.33	0.28–8.40	4	2.93	1.37	0.37–3.50
Lung	162	2	2.83	0.71	0.09–2.55	15	12.3	1.22	0.68–2.01
Breast	170	5	11.5	0.43	0.14–1.01	36	39.4	0.91	0.64–1.27
Cervix	171	6	2.98	2.01	0.74–4.38	2	3.40	0.59	0.07–2.12
Prostate	177	2	1.00	2.00	0.24–7.22	15	14.3	1.05	0.59–1.73
Urinary organs, including bladder	181	1	1.36	0.74	0.02–4.10	10	6.65	1.50	0.72–2.77
Melanoma	190	2	4.02	0.50	0.06–1.80	6	8.65	0.69	0.25–1.51
Nervous system, including brain	193	4	2.73	1.47	0.40–3.75	7	5.73	1.22	0.49–2.52
Non Hodgkin’s lymphoma	200,202	2	1.37	1.46	0.18–5.27	7	4.89	1.43	0.58–2.95
Hodgkin’s lymphoma	201	0	0.69	0.00	0.00–5.35	1	0.62	1.61	0.04–8.99
Multiple myeloma	203	1	0.35	2.86	0.07–15.9	1	1.74	0.57	0.01–3.20
Leukaemia	204–205	2	0.90	2.22	0.27–8.03	3	2.69	1.12	0.23–3,26
Lymphohaematopoietic cancer	200–209	6	3.58	1.68	0.61–3.65	12	10.8	1.11	0.57–1.94
All sites	140–209	41	50.3	0.81	0.58–1.10	162	166	0.98	0.83–1.14

* The follow up period started 1976 for the workers from plant A.

**Table 5.** External (SIR) and internal (IRR) comparison of cancer incidence between exposure categories within the cohort, calculated by Poisson regression using data stratified for gender, age and calendar period. **

***Tumour site (ICD7)***	***Cases***	***Person-years***	***SIR***	***95% CI***	***IRR***	***95% CI***
All sites (140–209)						
0–0.13 ppm-years (n = 1,039)	67	25,707	0.87	0.68–1.11	1.00	-
0.14–0.21 ppm-years (n = 486)	56	13,833	0.99	0.74–1.28	1.37	0.95–1.97
≥0.22 ppm-years (n = 495)	70	14,509	1.00	0.78–1.26	1.34	0.94–1.90
Breast cancer (170) *						
0–0.13 ppm-years (n = 615)	10	15,763	0.52	0.25–0.96	1.00	-
0.14–0.21 ppm-years (n = 287)	14	8,245	1.06	0.58–1.78	2.76	1.20–6.33
≥0.22 ppm-years (n = 295)	17	8,874	1.12	0.65–1.79	3.55	1.58–7.93
Lymphohaematopoietic cancer (200–209)						
0–0.13 ppm-years (n = 1,039)	7	25,707	1.35	0.54–2.78	1.00	-
0.14–0.21 ppm-years (n = 486)	5	13,833	1.32	0.43–3.09	1.17	0.36–3.78
≥0.22 ppm-years (n = 495)	5	14,509	1.08	0.35–2.54	0.92	0.28–3.05

* Only female workers were included in the calculation;

** One case of lymphohaematopoietic cancer and 10 cases of all sites lost due to unknown cumulative exposure.

## Abbreviations:

EtO: Ethylene oxide;
O: Observed;
E: Expected;
IRR: Incidence Rate ratio;
SIR: Standardized Incidence Ratio;
SMR: Standardized Mortality Ratio;
95% CI: 95% confidence interval
